# Right ventricular fibrosis in adults with uncorrected secundum atrial septal defect and pulmonary hypertension: a cardiovascular magnetic resonance study with late gadolinium enhancement, native T1 and extracellular volume

**DOI:** 10.3389/fcvm.2024.1395382

**Published:** 2024-05-30

**Authors:** Fatwiadi Apulita Ginting Munte, Elen Elen, Olfi Lelya, Estu Rudiktyo, Radityo Prakoso, Oktavia Lilyasari

**Affiliations:** ^1^ Department of Cardiology and Vascular Medicine, Faculty of Medicine, University of Indonesia, National Cardiovascular Center Harapan Kita, Jakarta, Indonesia; ^2^ Division of Cardiovascular Imaging and Nuclear Cardiology, Department of Cardiology and Vascular Medicine, Faculty of Medicine, University of Indonesia, National Cardiovascular Center Harapan Kita, Jakarta, Indonesia; ^3^ Division of Pediatric Cardiology and Congenital Heart Disease, Department of Cardiology and Vascular Medicine, Faculty of Medicine, University of Indonesia, National Cardiovascular Center Harapan Kita, Jakarta, Indonesia; ^4^ Division of Echocardiography, Department of Cardiology and Vascular Medicine, Faculty of Medicine, University of Indonesia, National Cardiovascular Center Harapan Kita, Jakarta, Indonesia

**Keywords:** right ventricular fibrosis, secundum atrial septal defect, pulmonary hypertension, late gadolinium enhancement, native T1, extracellular volume

## Abstract

**Introduction:**

Right ventricular (RV) fibrosis represents both adaptive and maladaptive responses to the overloaded RV condition. Its role in pulmonary hypertension (PH) associated with secundum atrial septal defect (ASD), which is the most common adult congenital heart disease (CHD), remains poorly understood.

**Methods:**

We enrolled 65 participants aged ≥18 years old with uncorrected secundum ASD who had undergone clinically indicated right heart catheterization (RHC), divided into the non-PH group (*n* = 7), PH group (*n* = 42), and Eisenmenger syndrome (ES) group (*n* = 16). We conducted cardiovascular magnetic resonance (CMR) studies with late gadolinium enhancement (LGE) imaging, native T1 mapping, and extracellular volume (ECV) measurement to evaluate the extent and clinical correlates of RV fibrosis.

**Results:**

LGE was present in 94% of the population and 86% of the non-PH group, mostly located at the right ventricular insertion point (RVIP) regions. LGE in the septal and inferior RV region was predominantly observed in the ES group compared to the other groups (*p* = 0.031 and *p* < 0.001, respectively). The mean LGE scores in the ES and PH groups were significantly higher than those in the non-PH group (3.38 ± 0.96 vs. 2.74 ± 1.04 vs. 1.57 ± 0.79; *p* = 0.001). The ES and PH groups had significantly higher degrees of interstitial RV fibrosis compared to those in the non-PH group, indicated by native T1 (1,199.9 ± 68.9 ms vs. 1,131.4 ± 47.8 ms vs. 1,105.4 ± 44.0 ms; *p* < 0.001) and ECV (43.6 ± 6.6% vs. 39.5 ± 4.9% vs. 39.4 ± 5.8%; *p* = 0.037). Additionally, native T1 significantly correlated with pulmonary vascular resistance (*r* = 0.708, *p* < 0.001), RV ejection fraction (*r* = −0.468, *p* < 0.001) and peripheral oxygen saturation (*r* = −0.410, *p* = 0.001).

**Conclusion:**

In patients with uncorrected secundum ASD, RV fibrosis may occur before the development of PH and progressively intensify alongside the progression of PH severity. A higher degree of RV fibrosis, derived from CMR imaging, correlates with worse hemodynamics, RV dysfunction, and poorer clinical conditions.

## 1 Introduction

Atrial septal defect (ASD) is a congenital heart disease (CHD) characterized by a defect in the interatrial septum, which allows blood to bypass between the pulmonary and systemic circulations. ASD is the most common CHD found in adults, comprising 35% of all CHDs (1), with secundum ASD as the most prevalent type (2). Patients with ASD typically remain asymptomatic until adulthood due to the slow and gradual process of right ventricular (RV) remodeling caused by chronic volume overload due to left-to-right shunting (2, 3). Increased blood flow to the lungs leads to the remodeling of the pulmonary vasculature, resulting in elevated pulmonary vascular resistance (PVR) and increased pressure in the RV (3).

Pulmonary hypertension (PH), defined by a mean pulmonary arterial pressure (mPAP) > 20 mmHg, is a chronic and life-threatening condition characterized by a progressive vasculopathy of pulmonary arterioles, resulting in increased PVR and decreased RV function (4, 5), with RV failure being the leading cause of mortality (6). The role of RV fibrosis in the pathophysiology of PH is under investigation. Pressure overload in the RV imposes mechanical stress on the interstitial and cardiomyocytes, leading to increased collagen production and fibroblast proliferation (7–9). Alterations in this collagen network are an adaptive response to prevent RV dilatation. However, these changes can also become maladaptive, which further impairs the RV function (9).

PH condition may complicate various forms of CHD with pulmonary circulation overflow, including simple pre-tricuspid shunts such as ASD. Among patients with secundum ASD, 8% had PH condition, of whom 29% had developed Eisenmenger syndrome (ES), characterized by the reversal of the systemic-to-pulmonary shunt (10). ES represents the severe end of the spectrum of PH associated with CHD. Defect closure in patients with irreversible pulmonary vascular disease (PVD) or ES is usually discouraged and hazardous to patients since the RV will be unable to overcome the high pulmonary resistance and will decompensate. It appears that closure beyond the “point of no return” relates to accelerated disease progression, as these patients have a prognosis substantially worse than those with uncorrected lesions (11).

Currently, cardiovascular magnetic (CMR) imaging has emerged as the gold standard for non-invasive assessment of myocardial fibrosis using techniques such as late gadolinium enhancement (LGE) imaging and T1 mapping. LGE is used to evaluate focal myocardial fibrosis, while native T1 and extracellular volume (ECV) are novel methods for quantifying diffuse interstitial fibrosis (12, 13). Additionally, CMR has been used in ASD patients for anatomic and hemodynamic evaluations, including measurement of RV volumes and ejection fraction, assessment of pulmonary venous connection, and quantification of shunt flow (Qp/Qs ratio). However, current guidelines do not incorporate CMR into the decision-making process for defect closure in cases of associated PH but solely depend on right heart catheterization (RHC) results (2, 14).

Several studies have reported that in the PH population, the degree of RV fibrosis, as measured by CMR, correlated with pulmonary arterial hemodynamics, RV function and volume, and clinical adverse outcomes (15–21). However, these studies have largely been conducted on PH of mixed or other than CHD etiologies. PH associated with ASD presents a unique pathophysiology characterized by chronic volume and later pressure overload probably resulting in distinct RV remodeling (22). The role of RV fibrosis in the development of severe PVD and RV failure in this population remains poorly understood.

Studies on the adult CHD population hypothesized that the presence of myocardial fibrosis is responsible for the persistent limitation in cardiovascular function after surgical repair of anatomic defects (23–26). Several CMR studies with LGE have reported macroscopic fibrosis in the RV of adult CHD, mostly with systemic RV and cyanosis (27–30). Identifying the “non-viable RV” is important to predict the likelihood of a defect closure having clinically relevant benefits. RV fibrosis measured by CMR may serve this role by providing information on whether irreversible cellular damage has occurred, thereby contraindicating the shunt closure (30).

Thus, our study demonstrated the presence and evaluated the extent of RV fibrosis obtained using CMR and its correlation with hemodynamic, functional, and clinical parameters. Furthermore, we compared LGE findings and native T1 and ECV mapping of the RV among different severities of PH in adults with uncorrected ASD. This study may become an initial investigation to evaluate the role of RV fibrosis as a novel biomarker of disease severity and its potential impact on clinical decision-making in patients with ASD.

## 2 Methods

### 2.1 Study design

A total of 65 patients aged ≥18 years old with unrepaired secundum ASD who had undergone clinically indicated RHC at National Cardiovascular Center Harapan Kita, Jakarta, Indonesia, were enrolled in this study between March 2023 and December 2023. The study population was divided into three groups of subjects based on RHC results. The first group (*n* = 7) consisted of ASD patients with normal mPAP ≤20 mmHg. The second group consisted of ASD patients with PH or mPAP >20 mmHg (*n* = 42). The third group consisted of ASD patients with ES (*n* = 16). We further reclassified the study population into groups with contraindications to defect closure and those without contraindications. Defect closure is contraindicated in patients with PA systolic pressure exceeding two-thirds of systemic pressure, PVR greater than two-thirds of systemic resistance, and/or Eisenmenger physiology (14).

All participants underwent CMR within 6 months after RHC. The exclusion criteria included the presence of other CHD, significant valvular disease excluding tricuspid and pulmonary regurgitation, coronary artery disease, history of pulmonary embolism, chronic lung disease, autoimmune disease, myocarditis, cardiomyopathy, known hypersensitivity to contrast agent, implantable cardioverter defibrillator or pacemaker implantation, claustrophobia, or a glomerular filtration rate of <30 ml/min/1.73 m^2^. This study was approved by our institutional review board, and all subjects provided written informed consent.

### 2.2 Clinical data

We collected the clinical data from all study participants, including demographics, World Heart Organization (WHO) functional class, vital signs, body weight and height, laboratory data, electrocardiography (ECG), and echocardiography data, including left ventricular ejection fraction (LVEF), tricuspid annular plane systolic excursion (TAPSE), tricuspid regurgitation (TR) severity, and TR max pressure gradient.

### 2.3 RHC examination

RHC was performed in all participants as part of their diagnostic assessment at our catheterization laboratory prior to the CMR examination. Hemodynamic calculations for ASD patients in our center used Fick's method, allowing the determination of both systemic and pulmonary cardiac output (CO) from pressure and oxygen saturation. The RHC parameters obtained included mean pulmonary artery pressure (mPAP), flow ratio (FR) or Qp/Qs, PVR, and pulmonary vascular resistance/systemic vascular resistance (PVR/SVR). PH is defined as mPAP >20 mmHg (5). ES is a condition characterized by the reversal of systemic-to-pulmonary shunt to bidirectional or reversed shunt, defined hemodynamically as PVR/SVR ≥ 1, PVRi > 10 WU m^2^, and FR < 1 (31).

### 2.4 CMR protocol

All CMR studies were performed using a standardized protocol with a 1.5 T MR scanner (SIGNA Voyager, GE HealthCare, Waukesha, WI, USA) with a 21-anterior channel and 32-posterior channel array for image acquisition (32). Patients were scanned in the supine position with retrospective ECG gating. Hematocrit and creatinine were obtained from each patient prior to the CMR scan.

Cine images were obtained with balanced steady-state free precession (bSSFP) sequences in short- and long-axis views. The acquisition parameters were as follows: echo time, 1.1 ms; repetition time, 3.0 ms; flip angle, 50°; array coil spatial sensitivity encoding technique (ASSET) with an acceleration factor of 2; slice thickness, 6 mm; spatial resolution, 1.9 × 1.9 mm^2^; and temporal resolution, 36 ms. Imaging was performed during suspended inspiration.

The contrast agent used was gadoterate meglumine (Dotarem, Guerbet LLC; Princeton, NJ, USA), administered at a dosage of 0.1–0.2 mmol/kg via an antecubital vein, followed by a 20 ml flush of 0.9% NaCl solution. LGE imaging with phase-sensitive myocardial delayed enhancement (PS MDE) was performed using fast gradient-echo (FGRE) sequences and 10 min post-contrast injection in short- and long-axis views (33). The protocol parameters were as follows: echo time, 1.5 ms; repetition time, 3.6 ms; flip angle, 45°; acceleration factor, 2; inversion time, 250–350 ms; slice thickness, 6 mm; spatial resolution, 1.9 × 2.1 mm^2^; and temporal resolution, 208 ms. The image was acquired at the end-diastolic period.

Native T1 mapping was performed using a modified Look–Locker inversion recovery (MOLLI) sequence with a 5(3)3 breath-hold scheme in basal, mid, and apical short-axis slices. The protocol parameters were as follows: echo time, 1.7 ms; repetition time, 3.9 ms; flip angle, 35°; acceleration factor, 2; slice thickness, 6 mm; spatial resolution, 1.9 × 2.6 mm^2^; temporal resolution, 278 ms; initial T1, 100 ms; and T1 increment, 80 ms. Post-contrast T1 mapping was performed using a MOLLI sequence with a 4(1)3(1)2 short T1 breath-hold scheme, conducted 10–30 min after intravenous contrast injection. The image was captured at the end-systolic phase with motion correction.

All examination protocols were performed by two experienced radiographers who were blinded to the study. CMR images were analyzed and interpreted using the CVI42 software package (Circle Cardiovascular Imaging, Calgary, AB, Canada) by two experienced cardiologists who specialized in cardiovascular imaging and were blinded to the RHC results. In 20 randomly selected patients, image analysis was repeated at least 1 month later by the same reader and by another different reader to determine the intra- and interobserver variability.

### 2.5 Chamber quantification

We measured RV and LV end-diastolic volumes (EDVs), end-systolic volumes (ESVs), and ejection fractions (EFs) derived from a stack of short-axis cine images in all patients. CMR images were analyzed semiautomatically followed by manual correction of the endocardial and epicardial contour. Papillary muscles, moderator bands, and trabeculations were considered as intracavitary lumens of ventricles. Systolic and diastolic left ventricular eccentricity index (LVEI) was defined as the ratio of the distance between the anterior–posterior wall and the septal–lateral wall of the LV, measured in midventricular short-axis view of cine imaging (34). The main pulmonary artery diameter was calculated by measuring the transverse diameter of the main pulmonary trunk in axial view cine images before branching into the left and right pulmonary arteries.

### 2.6 LGE

**Figure 1:**
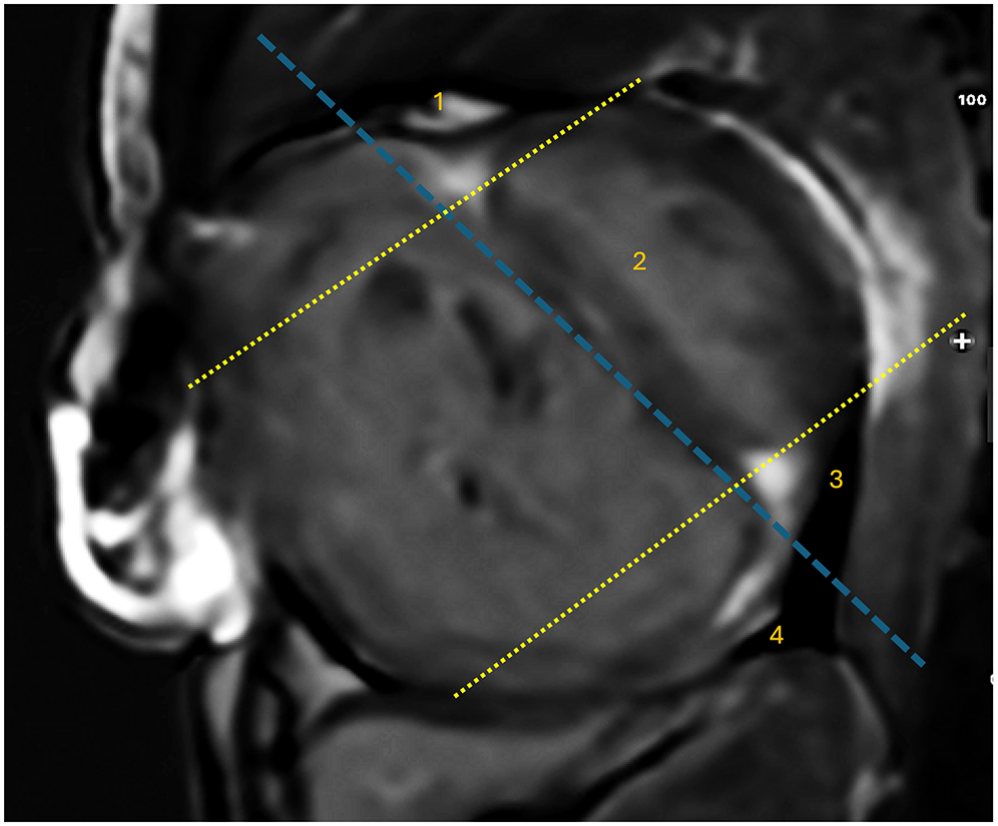
The locations of LGE corresponding to specific regions. There are four LGE locations identified: (1) anterior right ventricular insertion point (RVIP), (2) interventricular septum, (3) posterior RVIP, and (4) inferior RV free wall. These regions are demarcated by a dashed blue line parallel to the endocardial border of the interventricular septum on the RV side and a dashed yellow line parallel to the anterior and posterior endocardial borders on the LV side.

To evaluate the LGE extent, the presence of LGE was semiquantitatively assessed in four specific regions, including the anterior right ventricular insertion point (RVIP), interventricular septum (IVS), posterior RVIP, and inferior RV free wall (Figure 1). The midventricular short-axis slice served as the primary and representative view for the LGE analysis, with confirmation of its presence in other views. LGE was considered present if there was a bright signal within the darker normal myocardium in the midventricular slice and was also evident in either a more apical or basal view. The number of regions with LGE positive was summed in each patient, with a total score ranging from 0 to 4 (15, 17, 34).

### 2.7 Native T1 and ECV

**Figure 2:**
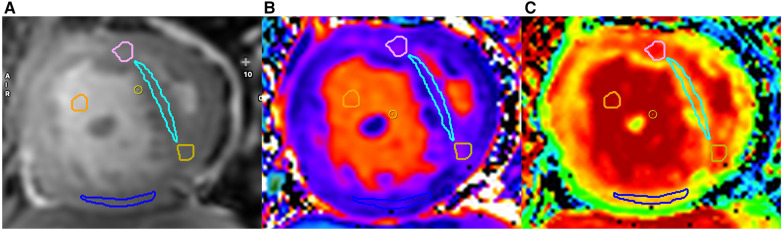
Examples of ROI locations obtained on cine images (**A**), native T1 (**B**), and ECV map (**C**) are shown. Each ROI from every region is distinguished by a different color: anterior right ventricular insertion point (RVIP) (pink), interventricular septum (light blue), posterior RVIP (yellow), inferior wall of the RV (blue), and blood pool (orange).

Native T1 measurement was obtained by manually drawing regions of interest (ROIs) in four locations on the midventricular short-axis plane including the anterior RVIP, IVS, posterior RVIP, and inferior RV free wall (Figure 2). ROIs were drawn with a minimum size of 12 pixels in each location and blinded to LGE findings. ROIs were carefully drawn on dense myocardial tissue without including non-myocardial structures such as trabeculae, epicardial fat, and blood pool. Native T1 values from each ROI location were recorded and averaged from four ROI locations.

Post-contrast T1 RV measurements were obtained on post-contrast maps using ROIs at the same locations as native T1 measurements. ECV calculation involved acquiring pre- and post-contrast myocardial T1 values at the same locations. Blood pool T1 values before and after contrast administration, along with hematocrit values, were also used for ECV calculation according to the formula and calculated automatically by the CVI42 program.

### 2.8 Statistical analysis

All statistical analyses were performed using the SPSS Statistics, Version 22.0 (IBM, NY, USA). Numeric data were expressed as mean ± standard deviation (SD), while categorical data will be presented as frequencies (*n*) and percentages (%). Normality tests such as Kolmogorov–Smirnov or Shapiro–Wilk were conducted to determine whether the data are normally distributed. The Levene test was used for the homogeneity of variance test.

The characteristics between subgroups were compared based on the type of data variable. The chi-square and Mann–Whitney *U*-tests were used to compare 2 × 3 categorical data. For numerical variables, a one-way ANOVA test was used for normally distributed data, and the Kruskal–Wallis test was used for non-normally distributed data. Either the Bonferroni test or Games-Howell test was used for *post hoc* correction. Pearson's correlation coefficient was used for correlation analyses in normally distributed data and Spearman correlation for non-normally distributed data. A *p*-value of <0.05 was considered to indicate statistically significant.

Intra- and interobserver variability was assessed for LGE score, native T1, and ECV in 20 randomly selected participants. The intraclass correlation coefficient (ICC) was reported as the primary measure of intra- and interobserver variability.

## 3 Results

### 3.1 Clinical characteristics

A total of 65 patients underwent CMR studies and were subsequently included in the analysis. The participants had a mean age of 33.9 ± 10.5 years (range, 19–60 years), predominantly females (80%), and mostly had WHO functional class II symptoms (66%). Demographic, clinical, and RHC characteristics of all participants are summarized in Table 1.

**Table 1:** Demographic, clinical, and RHC characteristics.

Variables	All (*n* = 65)	Non-PH (*n* = 7)	PH (*n* = 42)	ES (*n* = 16)	*p*-value
Age, yrs	33.9 ± 10.5	43.9 ± 16.5	33.4 ± 9.4	30.6 ± 7.7	0.126
Females, *n* (%)	52 (80)	4 (57.1)	33 (78.6)	15 (93.8)	0.046
WHO FC ≥III, *n* (%)	12 (18.5)	0 (0)	4 (9.5)	8 (50.0)	<0.001
Hypertension, *n* (%)	3 (4.6)	1 (14.3)	2 (4.9)	0 (0)	0.168
SpO2, %	93.1 ± 7.5	97.9 ± 1.2	94.5 ± 5.6	87.4 ± 10.1	0.003^†‡§^
SBP, mmHg	116.5 ± 15.0	126.7 ± 17.8	114.5 ± 14.4	117.1 ± 14.3	0.202
HR, bpm	80.3 ± 13.6,	74.6 ± 14.1	80.7 ± 12.7	81.8 ± 15.9	0.485
BMI, kg/m^2^	20.1 ± 4.6	21.1 ± 4.4	20.6 ± 4.4	18.3 ± 4.9	0.044^§^
CMR-RHC interval, days	41.8 ± 27.0	41.4 ± 11.7	41.4 ± 29.2	43.0 ± 27.2	0.813
ECG, *n* (%)					
AF or AFL	6 (9.2)	1 (14.3)	6 (14.3)	0 (0)	0.161
RAD or SAD	57 (87.7)	5 (71.4)	36 (85.7)	16 (100)	0.045
cRBBB	26 (60)	1 (14.3)	20 (47.6)	5 (31.3)	0.956
RV strain	44 (67.7)	0 (0)	29 (69.0)	15 (93.8)	<0.001
Laboratory					
Hematocrit, %	43.9 ± 6.6	40.8 ± 3.3	43.2 ± 6.1	47.1 ± 8.0	0.065
Creatinine, mg/dl	0.7 ± 0.2	0.8 ± 0.2	0.7 ± 0.2	0.7 ± 0.1	0.718
Echocardiography					
ASD diameter, mm	28.4 ± 9.4	29.1 ± 11.0	30.6 ± 9.3	22.3 ± 6.4,	0.009^§^
LVEF, %	67.5 ± 10.3	60.1 ± 6.8	67.2 ± 10.4	71.3 ± 9.8	0.055
TAPSE, mm	22.3 ± 6.8	27.7 ± 5.9,	22.7 ± 7.1	18.8 ± 4.2,	0.022^‡§^
TR max PG, mmHg	70.9 ± 29.5	40.3 ± 11.9	69.4 ± 29.8	88.4 ± 21.3	<0.001^†‡^
Significant TR, *n* (%)	43 (66.2)	2 (28.6)	29 (69.0)	12 (75.0)	0.084
RHC					
mPAP, mmHg	48.1 ± 19.7	16.9 ± 2.5	47.1 ± 17.0	64.3 ± 11.0	<0.001^†‡§^
Qp/Qs	1.9 ± 1.4	2.3 ± 0.5	2.3 ± 1.5	0.7 ± 0.2	<0.001^‡§^
PVR, WU	12.8 ± 12.3	1.1 ± 0.8	9.3 ± 7.2	27.1 ± 13.8	<0.001^†‡§^
PVRi, WU m^2^	17.5 ± 15.2	1.6 ± 1.2	13.7 ± 10.3	34.5 ± 14.9,	<0.001^†‡§^
PVR/SVR	0.6 ± 0.5	0.06 ± 0.03	0.37 ± 0.30	1.29 ± 0.21	<0.001^†‡§^
Contraindicated to closure, *n* (%)	30 (46.2)	0 (0)	14 (33.3)	16 (100)	<0.001
CMR					
RVEDVi, ml/m^2^	185.2 ± 72.0	162.3 ± 53.4	196.9 ± 82.6	164.2 ± 39.5	0.216
RVESVi, ml/m^2^	114.0 ± 52.1	67.2 ± 19.7	121.0 ± 57.0	116.1 ± 37.1	0.015^†‡^
RVEF, %	38.7 ± 12.7	58.2 ± 4.3	38.5 ± 11.4	30.8 ± 8.8	<0.001^†‡§^
LVEDVi, ml/m^2^	61.2 ± 17.5	51.7 ± 4.8	63.0 ± 20.0	60.7 ± 12.1	0.124
LVESVi, ml/m^2^	22.7 ± 13.7	16.1 ± 3.4	24.2 ± 15.9	21.8 ± 8.6	0.272
LVEF, %	64.5 ± 10.4	68.9 ± 6.0	63.5 ± 11.6	65.1 ± 8.2	0.544
MPA diameter (mm)	40.7 ± 7.2	33.4 ± 5.0	42.4 ± 7.0	39.3 ± 6.3	0.005^†^
Systolic LVEI	2.6 ± 1.0	1.39 ± 0.34	2.60 ± 0.89	3.07 ± 1.08	<0.001^†‡^
Diastolic LVEI	1.5 ± 0.3	1.30 ± 0.20	1.56 ± 0.23	1.64 ± 0.31	0.014^†‡^

Values are mean ± SD or *n* (%).

^†^*p* < 0.05 after Bonferroni or Games-Howell correction when the non-PH group was compared to the PH group.

^‡^*p* < 0.05 after Bonferroni or Games-Howell correction when the non-PH group was compared to the ES group.

^§^*p* < 0.05 after Bonferroni or Games-Howell correction when the PH group was compared to the ES group.

The ES group had a higher proportion of women, poorer WHO functional class, lower peripheral oxygen saturation, and lower body mass index compared to the other groups. Pulmonary hemodynamics assessed via RHC were significantly worse in the ES and PH groups compared to the non-PH group (*p* < 0.001). RV ejection fraction calculated from CMR volumetric were significantly lower in the ES and PH groups compared to the non-PH group (*p* < 0.001). The ES and PH groups also demonstrated a higher systolic eccentricity index compared to the non-PH group, suggesting a more severe cardiac deformity (*p* < 0.001).

### 3.2 RV fibrosis in different groups

**Figure 3:**
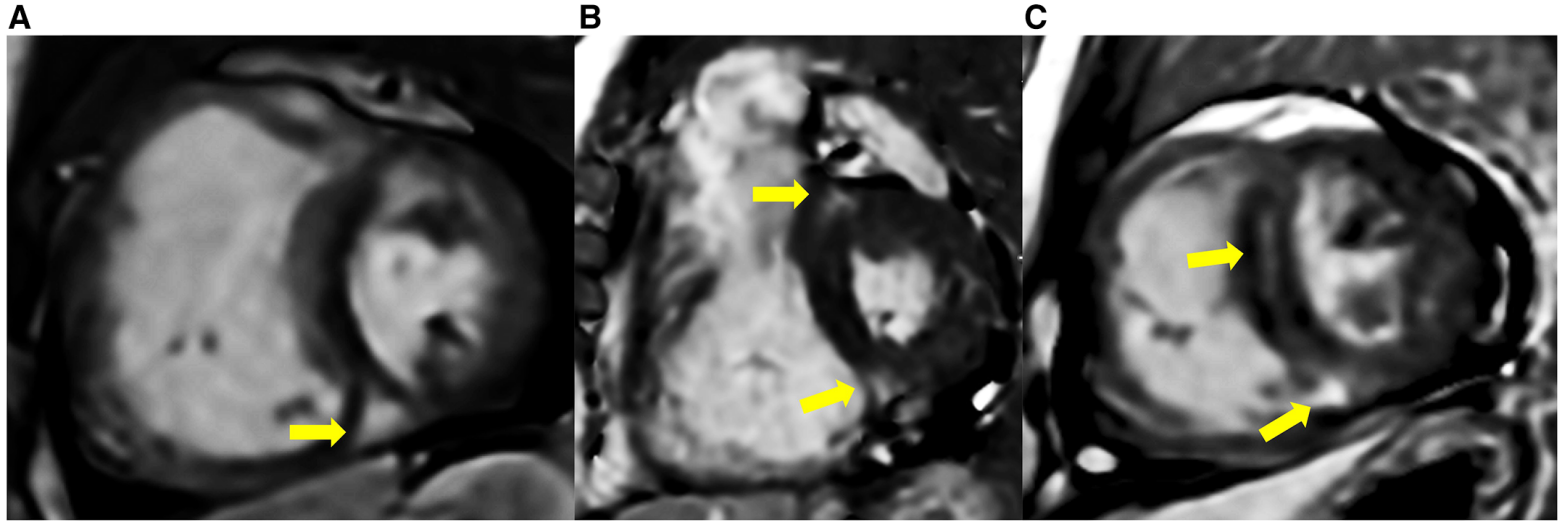
LGE was present in some ASD patients without PH, located at RVIPs and septal region (yellow arrow). (**A**) A 51-year-old male with mPAP 0f 18 mmHg, PVRi of 2.2 WU m^2^, Qp/Qs of 2.6, RVEF of 56%, and SpO2 of 98%. (**B**) A 64-year-old female with mPAP of 18 mmHg, PVRi of 3.8 WU m^2^, Qp/Qs 1.6, RVEF of 62%, and SpO2 of 97%. (**C**) A 51-year-old male with mPAP of 17 mmHg, PARi of 2.0 WU m^2^, Qp/Qs of 1.8, RVEF of 54%, and SpO2 of 99%.

**Figure 4:**
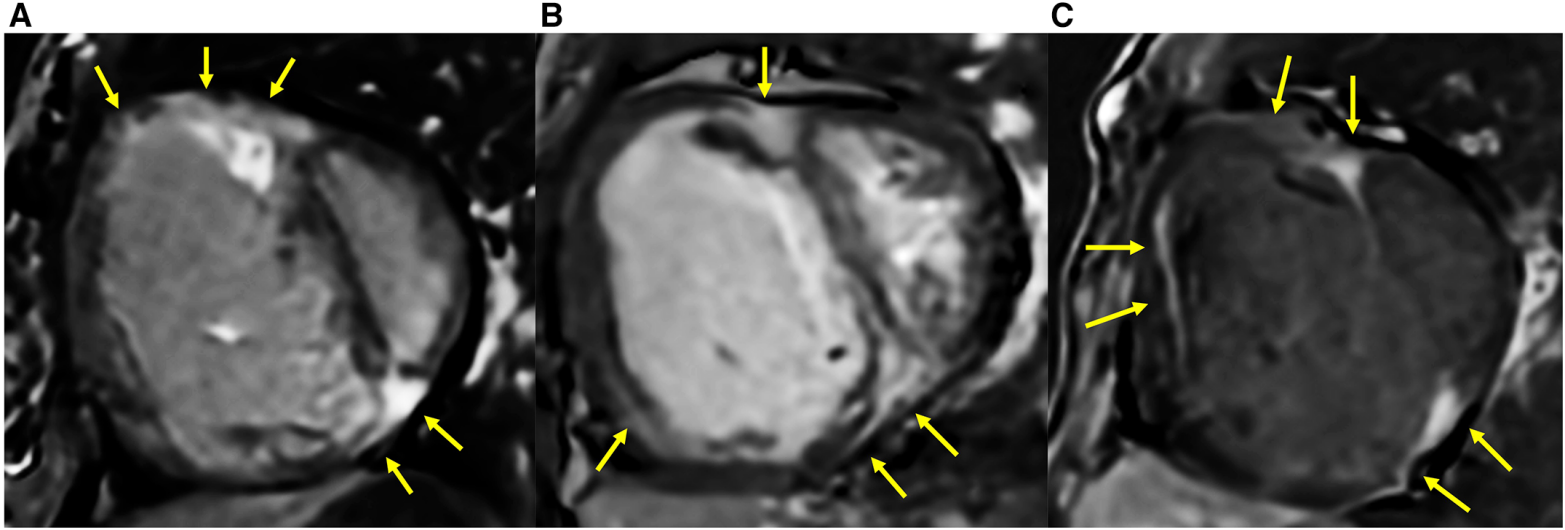
LGE extended into the inferior RV and other parts of the RV free wall in ASD patients who had developed Eisenmenger syndrome (yellow arrow). (**A**) A 25-year-old female with mPAP of 70 mmHg, PVRi of 55.4 WU m^2^, PVR/SVR 1.5, Qp/Qs of 0.5, RVEF of 30%, systolic eccentricity index of 2.95, and SpO2 77%. (**B**) A 29-year-old female with mPAP of 48 mmHg, PVRi of 16.3 WU m^2^, PVR/SVR 1.1, Qp/Qs of 0.7, RVEF of 28%, systolic eccentricity index of 2.02, and SpO2 of 94%. (**C**) A 39-year-old female with mPAP of 65 mmHg, PVRi of 30.2 WU m^2^, PVR/SVR of 1.03, Qp/Qs of 0.8, RVEF of 18%, systolic eccentricity index of 3.8, and SpO2 of 70%.

**Figure 5:**
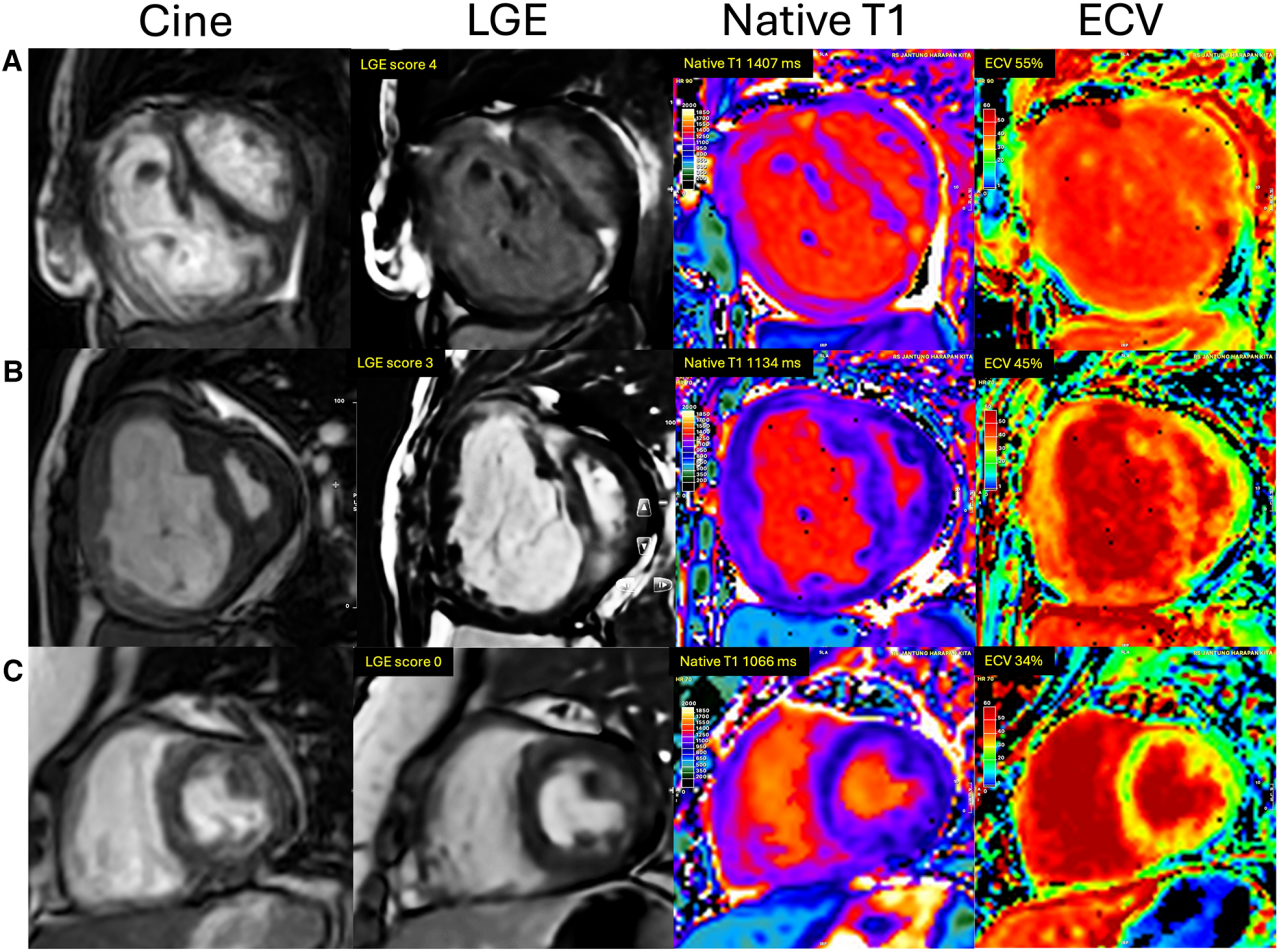
Examples of cine, LGE, native T1, and ECV image comparison between groups of ASD patients. (**A**) ES group: A 51-year-old woman with Eisenmenger syndrome had LGE at RVIPs extending to the septum and inferior RV, LGE score of 4, native T1 of 1,407 ms, and ECV of 55%. (**B**) PH group: A 26-year-old female with mPAP of 73 mmHg had an LGE score of 3, native T1 of 1,134 ms, and ECV of 45%. (**C**) Non-PH group: A 36-year-old woman with mPAP of 12 mmHg, no evidence of LGE, native T1 of 1,066 ms, and ECV of 34%.

The comparison of RV fibrosis parameters between groups is listed in Table 2. The presence of LGE was identified in 94% of the population and 86% of the non-PH group (Figure 3), mostly located in the RVIP region. LGE in the septal and inferior RV region was predominantly observed in the ES group compared to the other groups (*p* = 0.031 and *p* < 0.001, respectively). Examples of LGE images from patients with ES are depicted in Figure 4. The mean LGE scores in the ES and PH groups were significantly higher than those in the non-PH group (*p* = 0.001). In terms of interstitial fibrosis, the ES group exhibited significantly higher native T1 and ECV values compared to those of the other groups (*p* < 0.001 and *p* = 0.037, respectively), predominantly occurring in the RVIP region. Examples of native T1 and ECV mapping for each group compared to cine and LGE images are shown in Figure 5.

**Table 2:** RV fibrosis parameters in different groups.

Variables	All (*n* = 65)	Non-PH (*n* = 7)	PH (*n* = 42)	ES (*n* = 16)	*p*-value
Native T1 (average), ms	1,145.5 ± 61.7	1,105.4 ± 44.0	1,131.4 ± 47.8	1,199.9 ± 68.9	<0.001^‡§^
Anterior RVIP, ms	1,163.5 ± 91.6	1,087.4 ± 65.6	1,146.6 ± 71.3	1,241.2 ± 101.4	<0.001^‡§^
Septum, ms	1,114.8 ± 69.4	1,085.7 ± 53.2	1,106.5 ± 68.0	1,149.3 ± 70.2	0.053
Posterior RVIP, ms	1,184.8 ± 91.2	1,140.1 ± 109.0	1,165.2 ± 65.6	1,255.6 ± 107.8	0.001^‡§^
Inferior RV, ms	1,118.7 ± 66.3	1,108.1 ± 26.6	1,107.2 ± 72.7	1,153.6 ± 47.6	0.050
ECV (average), %	40.5 ± 5.7	39.4 ± 5.8	39.5 ± 4.9	43.6 ± 6.6	0.037^§^
Anterior RVIP, %	42.2 ± 6.9	41.3 ± 6.7	41.1 ± 6.5	45.4 ± 7.2	0.097
Septum, %	38.5 ± 7.0	37.4 ± 7.6	37.7 ± 6.3	40.9 ± 8.2	0.276
Posterior RVIP, %	42.2 ± 8.1	38.7 ± 10.1	41.0 ± 7.3	46.9 ± 7.7	0.018^§^
Inferior RV, %	39.2 ± 6.1	40.3 ± 4.0	38.2 ± 5.6	41.3 ± 7.9	0.209
Presence of LGE, *n* (%)	61 (93.8)	6 (85.7)	39.4 (92.9)	16 (100)	0.170
Anterior RVIP, *n* (%)	56 (86.2)	3 (42.9)	38 (90.5)	15 (93.8)	0.014
Septum, *n* (%)	44 (67.7)	2 (28.6)	29 (69.0)	13 (81.3)	0.031
Posterior RVIP, *n* (%)	60 (92.3)	6 (85.7)	38 (90.5)	16 (100)	0.171
Inferior RV, *n* (%)	20 (30.8)	0 (0)	10 (23.8)	10 (62.5)	0.001
LGE score	2.8 ± 1.1	1.57 ± 0.79	2.74 ± 1.04	3.38 ± 0.96	0.001^†‡^

Values are mean ± SD or *n* (%).

^†^*p* < 0.05 after Bonferroni or Games-Howell correction when the non-PH group was compared to the PH group.

^‡^*p* < 0.05 after Bonferroni or Games-Howell correction when the non-PH group was compared to the ES group.

^§^*p* < 0.05 after Bonferroni or Games-Howell correction when the PH group was compared to the ES group.

The characteristics of RV fibrosis indices based on the contraindication of ASD closure are summarized in Table 3. The patients with contraindications to defect closure showed significantly higher native T1 and ECV values (*p* < 0.001). LGE in the septal and inferior RV region was significantly more prevalent in the group with contraindications compared to those without contraindications (*p* = 0.002).

**Table 3:** RV fibrosis based on contraindication to defect closure.

Variables	Contraindication to defect closure		*p*-value
	Yes (*n* = 30)	No (*n* = 35)	
Native T1 (average), ms	1,183.7 ± 59.6	1,112.6 ± 41.7	<0.001
Anterior RVIP, ms	1,222.5 ± 84.2	1,113.0 ± 63.8	<0.001
Septum, ms	1,141.6 ± 61.6	1,091.9 ± 68.2	0.003
Posterior RVIP, ms	1,232.3 ± 90.0	1,144.0 ± 71.0	<0.001
Inferior RV, ms	1,138.6 ± 63.5	1,101.6 ± 64.6	0.024
ECV (average), %	43.5 ± 5.3	37.9 ± 4.6	<0.001
Anterior RVIP, %	45.8 ± 6.2	39.1 ± 5.9	<0.001
Septum, %	40.6 ± 6.9	36.6 ± 6.5	0.018
Posterior RVIP, %	47.0 ± 6.8	38.1 ± 6.8	<0.001
Inferior RV, %	40.7 ± 6.8	38.0 ± 5.2	0.077
Presence of LGE, *n* (%)	30 (100)	31 (88.6)	0.118
Anterior RVIP, *n* (%)	29 (96.7)	27 (77.1)	0.031
Septum, *n* (%)	26 (86.7)	18 (51.4)	0.002
Posterior RVIP, *n* (%)	30 (100)	30 (85.7)	0.057
Inferior RV, *n* (%)	15 (50)	5 (14.3)	0.002
LGE score	3.33 ± 0.80	2.29 ± 1.10	<0.001

Values are mean ± SD or *n* (%).

Using receiver operating characteristic (ROC) analysis, the cutoff value of native T1 to distinguish patients with contraindication to defect closure from ASD patients was found to be 1,145 ms, with a sensitivity of 83% and a specificity of 80% [area under the curve (AUC) = 0.864, 95% CI: 0.770–0.958, *p* < 0.001]. On the other hand, the cutoff value of ECV was 40.6%, with a sensitivity of 73% and a specificity of 74% (AUC = 0.782, 95% CI: 0.668–0.897, *p* < 0.001).

### 3.4 RV fibrosis correlates

The correlation between native T1 values, ECV values, and LGE score with hemodynamic and clinical parameters is detailed in Table 4. Native T1 values positively correlated with mPAP, PVR, PVR/SVR ratio, and RVESVi while negatively correlated with Qp/Qs, RVEF, and SpO2. Furthermore, the ECV values showed a positive correlation with mPAP, PVR, and PVR/SVR ratio and a negative correlation with Qp/Qs and SpO2. There were also significant correlations between the LGE score and other parameters, including mPAP, PVR, PVR/SVR ratio, Qp/Qs, RVEF, and SpO2.

**Table 4:** Correlation of RV fibrosis extent with various parameters.

Variables	mPAP		PVR		PVR/SVR		Qp/Qs		RVEDVi		RVESVi		RVEF		SpO2	
	*r*	*p*	*r*	*p*	*r*	*p*	*r*	*p*	*r*	*p*	*r*	*p*	*r*	*p*	*r*	*p*
Native T1	0.466*	<0.001	0.708*	<0.001	0.636*	<0.001	−0.595*	<0.001	0.055	0.665	0.348*	0.005	−0.468*	<0.001	−0.410*	0.001
Anterior RVIP	0.569*	<0.001	0.762*	<0.001	0.686*	<0.001	−0.617*	<0.001	0.010	0.938	0.335*	0.006	−0.550*	<0.001	−0.518*	<0.001
Septum	0.299*	0.015	0.406*	0.001	0.347*	0.005	−0.284*	0.022	0.201	0.109	0.376*	0.002	−0.330*	0.007	−0.176	0.162
Posterior RVIP	0.398*	0.001	0.626*	<0.001	0.598*	<0.001	−0.546*	<0.001	−0.023	0.856	0.216	0.084	−0.364*	0.003	−0.357*	0.004
Inferior RV	0.088	0.487	0.345*	0.005	0.331*	0.007	−0.438*	<0.001	0.012	0.925	0.064	0.624	−0.136	0.281	−0.242	0.052
ECV	0.290*	0.019	0.382*	0.002	0.398*	0.001	−0.455*	<0.001	−0.182	0.146	0.008	0.951	−0.231	0.064	−0.268*	0.031
Anterior RVIP	0.298*	0.016	0.400*	0.001	0.414*	0.001	−0.474*	<0.001	−0.149	0.237	−0.032	0.802	−0.237	0.058	−0.413*	0.001
Septum	0.155	0.217	0.175	0.164	0.221	0.077	−0.204	0.103	0.023	0.854	0.060	0.636	−0.097	0.440	−0.109	0.389
Posterior RVIP	0.454*	<0.001	0.514*	<0.001	0.507*	<0.001	−0.531*	<0.001	−0.197	0.116	0.035	0.783	−0.328*	0.008	−0.297*	0.016
Inferior RV	−0.026	0.836	0.048	0.706	0.095	0.452	−0.189	0.132	−0.100	0.428	−0.022	0.861	−0.076	0.549	−0.002	0.989
LGE score	0.519*	<0.001	0.615*	<0.001	0.572*	<0.001	−0.500*	<0.001	−0.044	0.726	0.184	0.143	−0.482*	<0.001	−0.446*	<0.001

*p < 0.05.

### 3.4 Intra- and interobserver agreement

We also assessed the intra- and interobserver reliability for native T1, ECV, and LGE score in 20 random patients. We found that the ICC for intraobserver agreement was 0.92 for native T1, 0.83 for ECV, and 0.95 for LGE score. For interobserver reliability, we found that the ICC was 0.88 for native T1, 0.85 for ECV, and 0.91 for LGE score.

## 4 Discussion

To the best of our knowledge, this is the first study that specifically investigated RV fibrosis using CMR in adults with uncorrected ASD. There are three major findings from this study: (1) RV fibrosis was present in most of the ASD population including in patients who had not developed PH; (2) ASD patients with PH and ES showed a higher extent of RV fibrosis compared to that in the non-PH group; and (3) RV fibrosis indices, as assessed by CMR, correlated with clinical and hemodynamic severity of PH and RV dysfunction.

LGE was found in nearly all patients of our study population (94%), including in patients without PH (86%). This is quite interesting, as it suggests that RV fibrosis in patients with ASD may occur before the development of pressure overload. Interatrial left-to-right shunts lead to pulmonary circulation and RV volume overload from a very early age. ASD patients with no PH showed no difference in RVEDVi and had relatively normal RV function compared to other groups. RV volume loading has been well tolerable for a long time, although radionuclide studies have detected delayed RV contraction in association with RV dilation even when RV ejection fraction was normal (35). A study with regional RV tissue Doppler imaging showed early relaxation abnormalities in ASD patients with long-standing volume overload (36). Taken together, this chronic volume overload likely leads to earlier RV remodeling in ASD patients even with normal pulmonary arterial pressure and RV function.

The most prevalent location of LGE findings was in the RVIP region, which is not very different from those in other PH populations (15–18). This finding was first identified in patients with PH by Blyth et al. (15) in 2005. It typically forms a triangular shape, with the basal part on the epicardial side and the apex directed toward the IVS (37). McCann et al. (16) subsequently found fibrosis in the RVIPs during the autopsy of two patients who died with severe PH. They hypothesized that this LGE represents the presence of focal fibrosis. The RVIP is known as a specific area where mechanical stress occurs even under normal physiological conditions, which is further exacerbated by increased RV pressure. An animal study using a hypobaric-hypoxic PH model also found that the RVIPs and later the septum are the earliest and most intensely affected areas by the increased RV pressure, indicated by elevated immunoreactive-atrial natriuretic peptide expression (38). This LGE at RVIPs is quite a common finding in RV with loading conditions and is not specific to any particular type of PH population (15–20).

In more severe PH cases, ASD patients demonstrated LGE extending into the septal region. Blyth et al. (15) also observed extensive LGE extending into the IVS region in PH patients with paradoxical septum motion. The high RV pressure leads to transeptal pressure toward the LV, resulting in an IVS paradoxical movement during the early diastolic phase. This septal bounce imposes additional wall stress not only on the RVIPs but also on the entire septum. Moreover, Swift et al. (39) also demonstrated that the extension of LGE into the septal region is a feature associated with poor outcomes, even though after adjusting for other factors, septal LGE is not an independent marker. The current study also showed in more extreme PVD conditions, such as ES, LGE extended into the RV free wall, including the inferior RV region. This unique pattern has not been reported in previous studies with other types of PH populations. Yamasaki et al. (34) conducted an LGE study on adults with various types of CHD and found extensive LGE in three ASD patients with ES, present in the areas including the RVIPs, IVS, and extending into the anterior and inferior parts of the RV free wall. The LGE extent in this study positively correlated with the systolic eccentricity index. The high eccentricity in ES patients, indicating a more extreme cardiac deformity due to RV overload, increases mechanical stress on the septal and RV free wall, which is associated with extensive myocardial fibrosis.

In regard to diffuse interstitial fibrosis, ASD patients who had developed PH exhibited higher native T1 and ECV values. This increase in native T1, especially at the RVIPs and septum, may be also related to the prior corresponding LGE findings, reflecting increased focal fibrosis in that area. However, Bull et al. (40) correlated native T1 values with histological findings and found a strong correlation between native T1 and collagen volume fraction in the tissue, indicating diffuse myocardial fibrosis. An experimental study on a chronic PH animal model also showed elevated native T1 and ECV values at the RVIPs, and histological examinations revealed increased interstitial collagen compared to the control group (41). As previously discussed about LGE, increased native T1 predilection at septum and insertion points might be affected by mechanical stress induced by septal motion, which is sign of RV overload. Unlike LGE, which is a dichotomous parameter requiring >15% collagen content (42), native T1 and ECV can identify small changes such as diffuse interstitial fibrosis before the appearance of LGE (43).

Native T1 and ECV in our study correlated well with RV hemodynamics and function, particularly at RVIP and septal regions. These findings are in good agreement with previous studies on the PH population, which found a moderate to strong correlation between native T1 at the insertion point and septal region with mPAP, PVR, and RV ejection fraction (43–46). Global RV and septal ECV were also shown to correlate to mPAP and RV function (21, 47). This correlation probably arises from the interrelation between RV overload, myocardial mechanics, and myocardial structure and geometry (45). Elevated native T1 and ECV values in the present study seem to be related to disease severity, suggesting a progressive and continuum process of RV fibrosis. The Eisenmenger group exhibited lower RV performance, worse functional class, and more hypoxemia. Previous studies on CHD patients showed volume and pressure overload due to left-to-right shunting lead to myocardial injury and subsequent irreversible myocardial remodeling, as evidenced by increased levels of troponin I (48). RV enlargement also increases myocardial oxygen demand, leading to relative myocardial hypoperfusion (3). Additionally, there is an increase in levels of amino-terminal procollagen type III peptide, reflecting collagen tissue synthesis, along with increased ventricular load and severity of cyanosis in the CHD population, including ASD (49).

The importance of our findings is not only in the demonstration of RV fibrosis and its extent but also in the potential application of CMR fibrosis indices as a tool for clinical decision-making. Current guidelines still use catheterization as the primary modality for evaluating ASD patients with PH, where the calculation of PVR is mandatory to decide whether ASD closure would be beneficial or harmful. However, some challenging cases fall into the “gray area” with marginal hemodynamics, making the decision for defect closure quite difficult (2, 14). Furthermore, a range of borderline cases may also be candidates for fenestrated closure. CMR fibrosis indices capable of detecting the presence of irreversible remodeling at the microscopic level of the RV seem suitable for aiding decisions regarding defect closure in such difficult cases. In our study, we identified the cutoff values of 1,145 ms for native T1 and 40.6% for ECV to distinguish ASD patients for whom defect closure is contraindicated. The presence of LGE at septal and inferior RV can also provide additional evidence to postpone ASD closure. Nevertheless, future prospective study is required to see if this CMR fibrosis index has significant clinical implications.

The serial measurement of RV fibrosis using CMR may be also beneficial in follow-up for reverse remodeling after an intervention. A systematic review and meta-analysis confirmed the consistent reduction in RV dimensions after ASD closure despite no significant improvement in RV function (50). Previous studies also showed that LGE at RVIPs completely resolved only in a small proportion of patients, while other patients had partial resolution or no change after ASD closure (51). Nonetheless, cardiac remodeling after ASD closure has not been thoroughly characterized using diffuse fibrosis indices such as T1 mapping. It would be interesting to see whether the normalization of the aforementioned elevated diffuse fibrosis indices, as measured by native T1 and ECV, occurs following defect closure.

## 5 Limitation

This study has some limitations. Firstly, this is a cross-sectional study with no follow-up; therefore, it could not determine the causal relationship between fibrosis and PH. The prognostic value of these RV fibrosis parameters remains unknown, necessitating further long-term study for evaluation. Secondly, the median interval between RHC and CMR examinations in our study was 37 days (4–139 days). This is due to the limited capacity of our CMR facility to accommodate the number of patients. Hemodynamic parameters such as mPAP may fluctuate during this period, although chronic fibrosis parameters such as LGE and T1 are unlikely to demonstrate significant variation (17). Nevertheless, a correlation analysis would be more representative if both examinations could ideally be performed within a 24 h interval. Thirdly, the presence of LGE and myocardial disarray in the RVIP region may still occur in a small proportion of healthy populations (52), warranting examinations on normal populations for comparison. Lastly, although various studies have shown that native T1 and ECV correlate well with histological fibrosis, it should be noted that besides fibrosis, increased values can also occur in other pathological conditions such as inflammation and edema.

## 6 Conclusions

In patients with uncorrected secundum ASD, RV fibrosis may occur before the development of PH and progressively intensify alongside the progression of PH severity. The patients with ASD who have developed PH and, to a greater extent, ES showed higher RV fibrosis indices, as measured by LGE imaging, native T1, and ECV. The degree of RV fibrosis also correlates with the severity of PH and RV dysfunction. In the future, RV fibrosis measured by CMR may become a valuable biomarker of severity and has a potential role in clinical decision-making such as defect closure.

## Data Availability

The raw data supporting the conclusions of this article will be made available by the authors, without undue reservation.
